# The pathological role of Wnt5a in psoriasis and psoriatic arthritis

**DOI:** 10.1111/jcmm.14531

**Published:** 2019-07-16

**Authors:** Faming Tian, Theodora M. Mauro, Zhengxiao Li

**Affiliations:** ^1^ Medical Research Center North China University of Science and Technology Tangshan China; ^2^ Dermatology Services Veterans Affair Medical Center and University of California‐San Francisco San Francisco CA USA; ^3^ Department of Dermatology The Second Affiliated Hospital of Xi'an Jiaotong University Xi'an China

**Keywords:** immunity, keratinocyte, psoriasis, psoriatic arthritis, vascularity, Wnt5a

## Abstract

Psoriasis (PsO) is a chronic inflammatory skin disease with both local and systemic components. PsO‐associated arthritis, known as psoriatic arthritis (PsA), develops in approximately 13%‐25% of PsO patients. Various factors associated with both PsO and PsA indicate that these conditions are part of a single disease. Identification of novel targets for the development of drugs to treat both PsO and PsA is desirable to provide more patient‐friendly treatment regimens. Such targets will likely represent ‘common checkpoints’ of inflammation, for example key components or transduction cascades of the signalling pathways involved. Emerging evidence supports involvement of the non‐canonical Wnt signalling pathways in the development of both PsO and PsA, especially the Wnt5a‐activated signalling cascades. These, together with interlinked factors, are crucial in the interactions among keratinocytes, immune cells and inflammatory factors in PsO, as well as among chondrocytes, osteoblasts and osteoclasts that trigger both subchondral bone remodelling and cartilage catabolism in PsA. This review focuses on the pathological role of Wnt5a signalling and its interaction with other interlinked pathways in both PsO and PsA, and also on the main challenges for future research, particularly with respect to molecules targeting Wnt signalling pathways for the treatment of PsO and PsA.

## 1 INTRODUCTION

Psoriasis (PsO) is a chronic inflammatory skin disease that affects up to 3.8% of the population.^1^ Cell‐mediated immunity, excessive growth and aberrant differentiation of keratinocytes, and increased dermal vascularity all play important roles in the pathomechanisms of PsO.^2^ Approximately 13%‐25% of PsO patients develop psoriatic arthritis (PsA), characterized by peripheral arthritis, axial spondylitis and enthesitis.^3^, ^4^ According to the Classification of Psoriatic Arthritis (CASPAR) criteria,^5^ current or past presence of psoriasis of the skin, or a positive family history, represents a major criterion for the diagnosis of PsA. Interaction among genetic, environmental and immune factors leads to psoriatic skin and joint manifestations.^6^, ^7^ Both the skin and synovium of patients with PsA produce increased concentrations of pro‐inflammatory cytokines. Various factors, including human leucocyte antigens (HLA), the interleukin (IL)‐23/IL‐17 axis and tumour necrosis factor‐α (TNF‐α), are related to PsO and PsA and support the hypothesis that PsO and PsA are different manifestations of a single disease.^8^, ^9^

On the one hand, revealing differences between PsO and PsA will provide insight into their respective pathophysiologies. On the other hand, identification of novel targets for treatment of both PsO and PsA is important for simpler and more patient‐friendly treatment regimens. These targets will most likely represent ‘common checkpoints’ of inflammation, including key components or transduction cascades of the signalling pathways involved, rather than ‘common denominators’ such as cytokines.^10^, ^11^ For example, small molecules that inhibit enzymes such as Janus kinases or phosphodiesterase 4 have proved effective in treating PsO and PsA.^12^, ^13^ In this context, the Wnt5a signalling pathway is an attractive target for the treatment of PsO as well as PsA.

## 2 WNT5A AND PSO

### 2.1 Wnt5a signalling pathway

**Figure 1:**
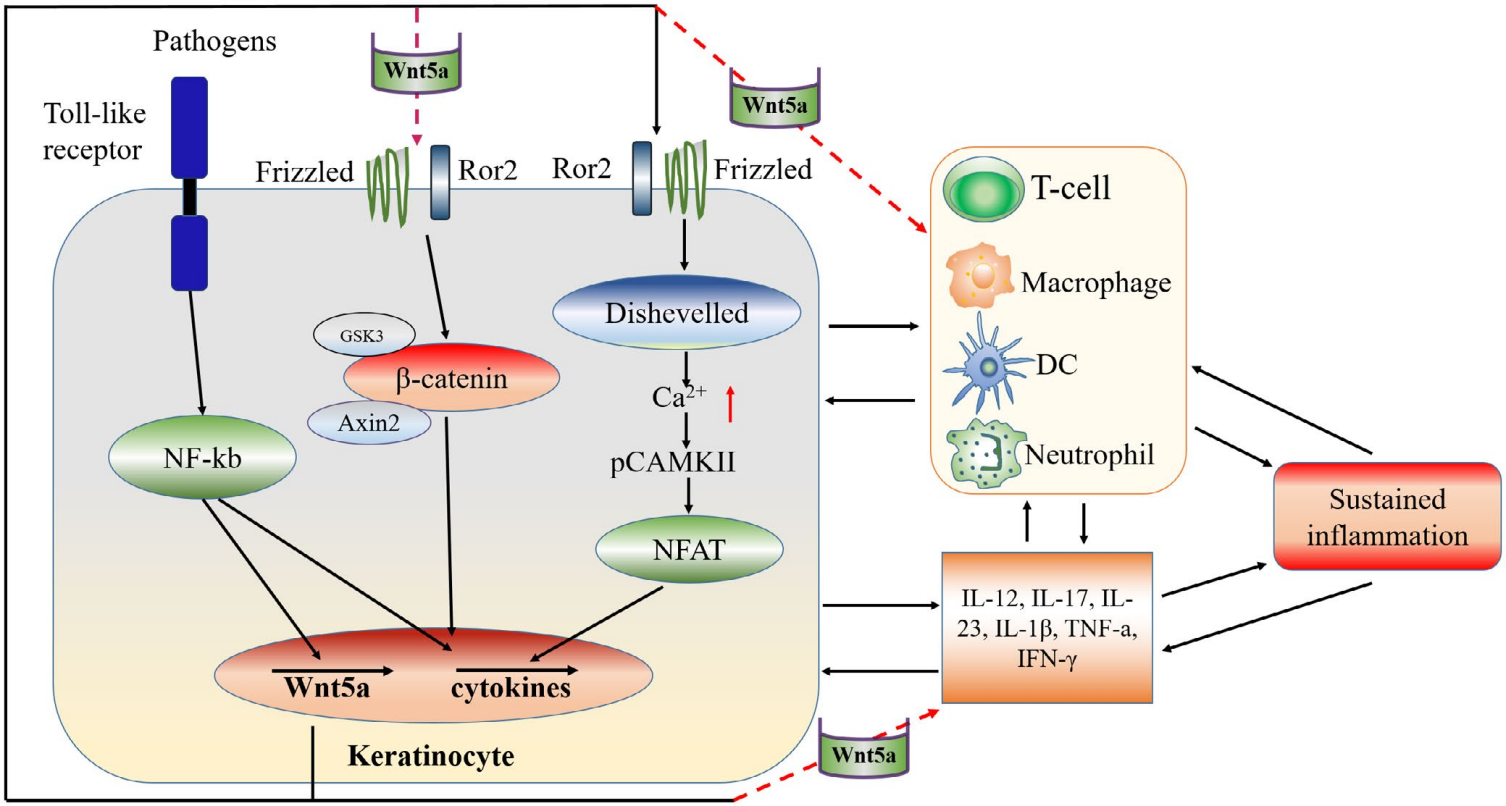
Model of the role and proposed mechanism of Wnt5a in psoriasis. Activation of Wnt5a signaling and its downstream effectors by local or systemic pathogens stimulate keratinocyte proliferation and secretion of inflammatory cytokines, which further regulate Wnt5a expression and promote keratinocyte proliferation and activation through Wnt5a‐mediated signalling pathways. This cross‐talk forms a signalling loop that promotes the persistence of PsO inflammation and disease progression

Wnt signalling, which plays important roles in regulating cell proliferation, differentiation, polarity, migration and inflammation,^14^, ^15^, ^16^, ^17^, ^18^ is classified into β‐catenin‐dependent canonical and β‐catenin‐independent non‐canonical pathways. In the canonical pathway, Wnt signalling is activated by Wnt proteins binding to their respective dimeric cell surface receptors composed of the seven‐transmembrane Frizzled proteins and the low‐density lipoprotein receptor‐related proteins (LRP5/6). Upon Wnt‐Fz/LRP signalling, Dvl is activated and dissociates from a multiprotein complex leading to inactivation of GSK3β. This inhibits the phosphorylation and degradation of β‐catenin, which accumulates in the cytoplasm and then translocates to the nucleus and interacts with lymphoid enhancer‐binding factors (LEF) and T cell factors (TCF), causing transcriptional activation of target genes^14^ (Figure 1).

The key molecules and cascades in the non‐canonical pathway have been previously summarized.^19^, ^20^ Briefly, non‐canonical Wnt signal transduction, predominantly of Wnt5a, mainly involves planar cell polarity (PCP) and Wnt/Ca^2+^ pathways. Non‐canonical Wnt signalling pathways, which are independent of β‐catenin, rely on Wnt signal transduction through Fzd and its coreceptors, such as receptor tyrosine kinase‐like orphan receptor 2 (ROR2) or receptor‐like tyrosine kinase (RYK). Through the activation of calcium signalling (phospholipase C/protein kinase C (PKC)/Ca^2+^) and calmodulin‐sensitive protein kinase II (CamkII), the Wnt/Ca^2+^/CamkII pathway activates nuclear factor associated with T cells (NFAT) to regulate cell adhesion and migration, as well as cytoskeletal rearrangements. In the PCP pathway, Wnt binds to frizzled (Fzd) receptors, activates dishevelled and thereafter triggers Rho/Rho‐associated kinase (ROCK), Rac/c‐Jun N‐terminal kinase (JNK) signalling and actin polymerization. These complex signalling events are integrated to mediate cytoskeletal changes, cell polarization and motility during gastrulation. (Figure 1) Recent evidence supports the involvement of Wnt5a in inflammatory diseases,^21^, ^22^ particularly in the development of psoriatic lesions.^23^, ^24^, ^25^, ^26^

### 2.2 Wnt5a is differentially expressed in psoriatic skin

Reischl et al^23^ found that Wnt5a expression was fourfold higher than normal in skin lesions from patients with plaque‐type psoriasis. Another study with more subjects showed that Wnt5a transcripts were up‐regulated fivefold in skin lesions and that FZD2 and FZD5 expression was also increased, while mRNA levels of WIF1 (a Wnt antagonist) were down‐regulated >10‐fold.^24^ We previously demonstrated overexpression of Wnt5a in PsO lesions, and in vitro analysis of Wnt5a knockdown in HaCaT and NHK cells suppressed cell proliferation and induced apoptosis.^25^ A recent analysis of gene pathogenicity using samples from psoriatic and healthy skin revealed that Wnt5a is one of five key genes in psoriasis.^26^ However, Wnt5a expression remains high in resolving psoriatic lesions^27^; therefore, the role of enhanced Wnt5a expression in PsO is more complicated than proposed. Mice with overexpression of Wnt5a in the epidermis do not exhibit a psoriasis phenotype.^28^

The correlation between epigenetic modifications and psoriasis was investigated by Verma et al^29^ They compared epidermis from PsO patients with that from healthy controls and identified more than 2000 strongly differentially methylated sites (DMS), including in Wnt5a and FZD2, as well as a notable overrepresentation of sites in genes of the cadherin and Wnt signalling pathways.^29^ NFATc1, a downstream gene of Wnt5a and important in imiquimod‐induced psoriasiform dermatitis,^30^ was differentially methylated at multiple sites, as was SFRP4, a negative regulator of Wnt signalling that is down‐regulated in PsO by an epigenetic mechanism.^31^

Together, the above findings show that Wnt5a is overexpressed in PsO lesions and probably plays an important role in PsO development. However, the degree to which Wnt5a up‐regulation is involved in the pathomechanism of psoriatic lesions remains unclear. Indeed, abnormal Wnt5a expression may actually counteract the primary defect to maintain a normal skin phenotype. Further studies are needed to clarify the role and mechanisms of Wnt5a in the complicated pathophysiology of PsO.

## 3 PROPOSED MECHANISM OF WNT5A INVOLVEMENT IN PSO

### 3.1 Wnt5a and keratinocytes

Interplay between the immune system and the epithelium is the pathological trigger of PsO. The activated adaptive and innate immune systems and T cell responses produce biochemical signals that stimulate keratinocyte hyperproliferation, interfere with their terminal differentiation and induce the secretion of pro‐inflammatory factors that, in turn, activate T cells. The activity and function of keratinocytes play determinant roles in PsO development and are key points that could be targeted by potential or emerging PsO treatments.

A series of in vitro studies has determined the effects of Wnt5a on keratinocytes. Treatment with recombinant Wnt5a increased human keratinocyte proliferation and secretion of TNF‐α, IL‐12 and IL‐23. IL‐1α, TNF‐α, transforming growth factor‐α and interferon‐γ stimulated keratinocytes to produce higher levels of Wnt5a, which, in turn, repressed both Notch1 and HES1.^32^ Knockdown of Wnt5a suppressed keratinocyte proliferation and induced apoptosis by repressing the Wnt5a/Ca^2+^ or Wnt/β‐catenin pathways.^25^ The calcium‐sensing receptor (CaSR) is essential in calcium‐induced differentiation of normal human epidermal keratinocytes (NHEKs) because it increases the level of free intracellular calcium, which up‐regulates the expression of Wnt5a. Subsequently, autocrine Wnt5a promotes the differentiation of NHEKs.^33^ In contrast, Wnt5a treatment can suppress HaCaT keratinocyte proliferation and differentiation, although the expression of IL‐8, IL‐17A and interferon‐γ was up‐regulated.^34^ It is difficult to interpret these somewhat contradictory results, but they indicate a complex and as yet unclear role of Wnt5a in modulating the proliferation and differentiation of keratinocytes.

Furthermore, these in vitro studies do not tell the whole story of what happens in vivo, where Wnt5a can be produced not only by keratinocytes, but also by other cells that participate in PsO development. To date, no studies have reported that an exogenous Wnt5a inhibitor or conditional deletion of Wnt5a from keratinocytes in vivo alters the development of PsO. Such studies are in progress in our laboratory.

### 3.2 Wnt5a and inflammation

#### 3.2.1 Interaction with inflammatory cytokines

Immune cell infiltration is one of the main characteristics of psoriatic lesions. As a potent signalling molecule, Wnt5a is strongly implicated in a number of inflammatory diseases, including PsO, rheumatoid arthritis and sepsis.^19^, ^35^, ^36^ Wnt5a triggers pro‐inflammatory signalling cascades and increases the expression levels pro‐inflammatory cytokines and chemokines. Conversely, Linnskog et al^37^ demonstrated a dose‐dependent increase in Wnt5a expression in IL‐6‐stimulated human melanoma cell lines, HTB63 and A375, whereas Box5, a peptide antagonist of Wnt5a, inhibited IL‐6‐induced cell migration and invasion of the melanoma.

Further study explored the regulatory effect of inflammatory cytokines on Wnt5a expression. Rauner et al^38^ found that TNF‐α could stimulate Wnt5a expression in human bone marrow stromal cells. IL‐17 is a target for PsO treatment but no report has focused on the interaction between Wnt5a and IL‐17 during PsO, although both are elevated in PsO lesions.^24^ However, stimulation of fibroblast‐like synoviocytes with TNF‐α and IL‐17A led to increased expression of Wnt5a.^39^ In vitro costimulation of mouse fibroblasts with purified IL‐17A and Wnt5a resulted in transforming growth factor‐β1 secretion and collagen transcription.^40^

These results indicate interplay between Wnt5a signalling and inflammatory responses, which may be dependent on Wnt5a‐mediated interaction with different leucocytes and keratinocytes. Accordingly, emerging evidence supports the regulatory roles of Wnt signalling pathways in leucocyte function.

#### 3.2.2 T cells

Wnt5a is a critical mediator of migration and CXC chemokine ligand‐12 (CXCL12)–CXC chemokine receptor‐4 (CXCR4) signalling in human and murine T cells. Levels of Wnt ligands are significantly increased in CXCL12‐treated T cells, while Wnt5a augments signalling through the CXCL12‐CXCR4 axis by activating PKC. Moreover, Wnt5a is essential for CXCL12‐mediated migration of T cells, and recombinant Wnt5a sensitizes human T cells to CXCL12‐mediated migration. Furthermore, Wnt5a is required for the sustained expression of CXCR4. These findings are supported by an in vivo study of T cell migration in EL4 thymoma metastasis.^41^

#### 3.2.3 Dendritic cells

Dendritic cells (DCs) functionally regulate immune responses by linking innate and adaptive immune systems.^42^ Accumulating evidence supports involvement of the Wnt signalling pathway in controlling immune balance via DCs. Increased Wnt5a signalling during DC differentiation compromises their functional capabilities. Wnt5a does not block the generation of DCs from monocytes but leads to phenotypically altered DCs that have a lower capacity to uptake antigens and that show an altered response to Toll‐like receptor (TLR) ligands. These effects are dependent on non‐canonical Ca^2+^/CamkII/NF‐κB signalling, indicating that Wnt5a skews human monocyte–derived DCs to differentiate into a tolerogenic functional state.^42^ Moreover, although both canonical and non‐canonical Wnts suppress murine DC pro‐inflammatory responses to bacterial endotoxin, IL‐6 production in DCs stimulated by the viral mimic, polyinosinic:polycytidylic acid, was inhibited by Wnt5a, but not Wnt3a.^43^ Holtzhausen et al^44^ demonstrated that Wnt5a promotes local dendritic cell expression of indoleamine 2,3‐dioxygenase‐1 (IDO) in a β‐catenin signalling pathway–dependent manner; Wnt5a‐conditioned DCs promote Treg cell differentiation in an IDO‐dependent manner.

In contrast, DCs isolated from the colon of Wnt5a‐ and receptor tyrosine kinase‐like orphan receptor 2 (Ror2)‐deficient mice impair the differentiation of naïve CD4^+^ T cells into interferon‐γ‐producing CD4^+^ Th1 cells. Furthermore, the Wnt5a‐Ror2 signalling axis augments the priming effect of DCs on interferon‐γ production, which subsequently enhances lipopolysaccharide (LPS)‐induced IL‐12 expression.^45^ The dual role of Wnt5a in DCs as a pro‐inflammatory and tolerogenic molecule indicates a complicated mechanism by which Wnt5a modulates DC differentiation and function. Wnt5a may modulate DC responses to limit inflammation, and its regulation of the immune response is a primordial mechanism for achieving immune homoeostasis.

#### 3.2.4 Macrophages and neutrophils

Macrophage recruitment is another characteristic of inflammation, including in PsO. An in vitro study focusing on Wnt5a interaction with macrophages in castration‐resistant prostate cancer (CRPC) indicated that Wnt5a may be a crucial regulator that induces CRPC in the bone niche by recruiting and regulating macrophages.^46^ Another in vitro study confirmed that Wnt5a induces macrophage chemotaxis and activation.^47^ Recombinant Wnt5a‐induced cytokine secretion by macrophages from C57BL/6 mice was dependent on TLR4 and was repressed by polymyxin B.^48^ Moreover, Wnt5a is up‐regulated in macrophages stimulated with endotoxin (LPS), which induces the expression of IL‐1β, IL‐6, IL‐8 and macrophage inflammatory protein‐1β.^22^ In fact, macrophage‐derived Wnt5a is an important regulator of macrophage immune function, pro‐inflammatory cytokine release, angiogenesis and lymphangiogenesis.^49^

Human neutrophils express a number of Wnt5a receptors, including FZD2, 5 and 8. Wnt5a stimulation of human neutrophils leads to chemotactic migration and the secretion of CXCL8 and CCL2. Neutrophil chemotaxis induced by supernatant collected from LPS‐stimulated macrophages was markedly inhibited by an antagonist of Wnt5a, which indicates that Wnt5a may contribute to neutrophil recruitment, thereby regulating the inflammation response.^50^

### 3.3 Wnt5a and angiogenesis

Dysregulated angiogenesis has been observed in the chronic cutaneous inflammation associated with PsO. Different angiogenic growth factors are involved at each step of the PsO molecular pathway, such as vascular endothelial growth factor, hypoxia inducible factor‐1α, and angiopoietin‐2.^51^, ^52^ The Wnt5a‐mediated non‐canonical signalling pathway potentially participates in this process, based on its important role in endothelial cell proliferation and vascularization.

Wnt5a is expressed in human primary endothelial cells, and exogenous Wnt5a expression in these cells promotes angiogenesis. Wnt5a induces endothelial cell proliferation and enhances cell survival by activating Ca^2+^/CamkII, whereas reduced Wnt5a expression decreases capillary‐like network formation and inhibits endothelial cell migration. Thus, Wnt5a promotes angiogenesis through non‐canonical pathways.^53^ In human vascular endothelial cells, Wnt5a regulates cytoskeleton remodelling and barrier function.^54^ Furthermore, Wnt5a can enhance the permeability of human coronary artery endothelial cells (HCAECs) through Ryk interaction and downstream ROCK/LIMK2/CFL1 signalling.^55^ Similarly, Wnt5a mediates remodelling of actin cytoskeleton in IL‐4‐activated HCAECs; silencing Wnt5a significantly reduced the enhanced permeability and improved barrier function in IL‐4‐treated HCAEC monolayers.^56^ In this context, Wnt5a may not only participate in angiogenesis by stimulating endothelial cell proliferation, but may also enhance the permeability of vascular endothelial cells, which is supposed to contribute to leucocyte effusion and infiltration in psoriatic lesions.

Taking these observations together, we present a model in which Wnt5a activation is involved in keratinocyte proliferation and secretion of inflammatory cytokines, which further regulate Wnt5a expression and promote keratinocyte proliferation and activation through Wnt5a‐mediated signalling pathways (Figure 1). This cross‐talk forms a signalling loop that promotes the persistence of PsO inflammation and disease progression.

## 4 WNT5A AND PSA

PsA targets the spine, peripheral joints and the entheses.^55^ The aetiology of PsA is unclear, but is thought to be an interplay of genetic, immunological and environmental factors that promote pathological bone remodelling and joint damage. Sixty‐seven per cent of PsA patients exhibit erosive bone disease,^56^ in which increased osteoclast activity causes destructive bone loss in both a localized and a systemic manner.^57^, ^58^, ^59^ The simultaneous presence of bone erosions and bony spurs in PsA joints indicates that PsA leads to activated bone remodelling with both enhanced bone resorption and bone formation. Abnormal bone remodelling therefore plays a crucial role in PsA.^60^

In contrast to PsO, in which Wnt5a is overexpressed, there has been no report of Wnt5a expression in PsA tissues, although Wnt5a is expressed locally in the joints of spondyloarthritis patients, which include PsA patients. Moreover, Wnt5a decreases differentiation marker gene expression and mineralization in cultured chondrocytes. It also decreases alkaline phosphatase activity in Achilles tendon enthesis and reduces osteocalcin levels released by ankle explants. In contrast, Wnt5a stimulates ossification marker expression in cultured osteoblasts and increases the tibial plateau bone volume in cultured explants of mouse ankle.^61^ Wnt5a is also involved in arthritis development by promoting osteoclast activity and the inflammation response.^62^ Wnt5a conditional knockout mice are resistant to the development of arthritis compared with control littermates, providing more insight into the role of endogenous Wnt5a in autoimmune diseases.^62^

Another in vitro study revealed a regulatory role of Wnt5a in osteoblasts and osteoclasts, which are the predominant cells in bone remodelling.^63^ Wnt5a expression was increased in osteoarthritic osteoblasts compared with their normal counterparts. Wnt5a increased the expression of LGR5 and stimulated the phosphorylation of JNK and PKC, and the activity of transcription factors NFAT and AP‐1. Inhibition of Wnt5a expression partially corrected the abnormal mineralization, osteocalcin secretion and ALPase activity of osteoarthritic osteoblasts.^63^

Moreover, osteoclastogenesis is enhanced by Wnt5a‐Ror2 signalling from osteoblast‐lineage cells to osteoclast precursors. Specifically, knockout of Wnt5a in osteoblasts or Ror2 in osteoclast precursors in mice caused reduced osteoclastogenesis.^64^ Wnt5a‐Ror2 signals increased the expression of receptor activator of nuclear factor‐κB (RANK) in osteoclast precursors by activating JNK and recruiting c‐Jun to promote the expression of RANK, thereby enhancing RANKL‐induced osteoclastogenesis. A soluble form of Ror2 acts as a decoy receptor for Wnt5a and abrogates bone destruction in mouse arthritis.^64^, ^65^ Similar results were found in other studies. Mice with an osteoclast‐specific deficiency in Ror2 had increased bone mass. Osteoclasts derived from these mice exhibited impaired bone resorption and actin ring formation.^66^ Wnt5a‐Ror2 signalling in the subchondral bone marrow stromal cells of temporomandibular joints, which was enhanced by experimentally induced unilateral anterior crossbite, promoted increased stromal cell expression of CXCL12 and RANKL. The JNK and/or Ca^2+^/NFAT pathways were involved and were therefore engaged in enhancing osteoclast precursor migration and differentiation, leading to increased osteoclast activity and overall subchondral trabecular bone loss in this model.^67^

**Figure 2:**
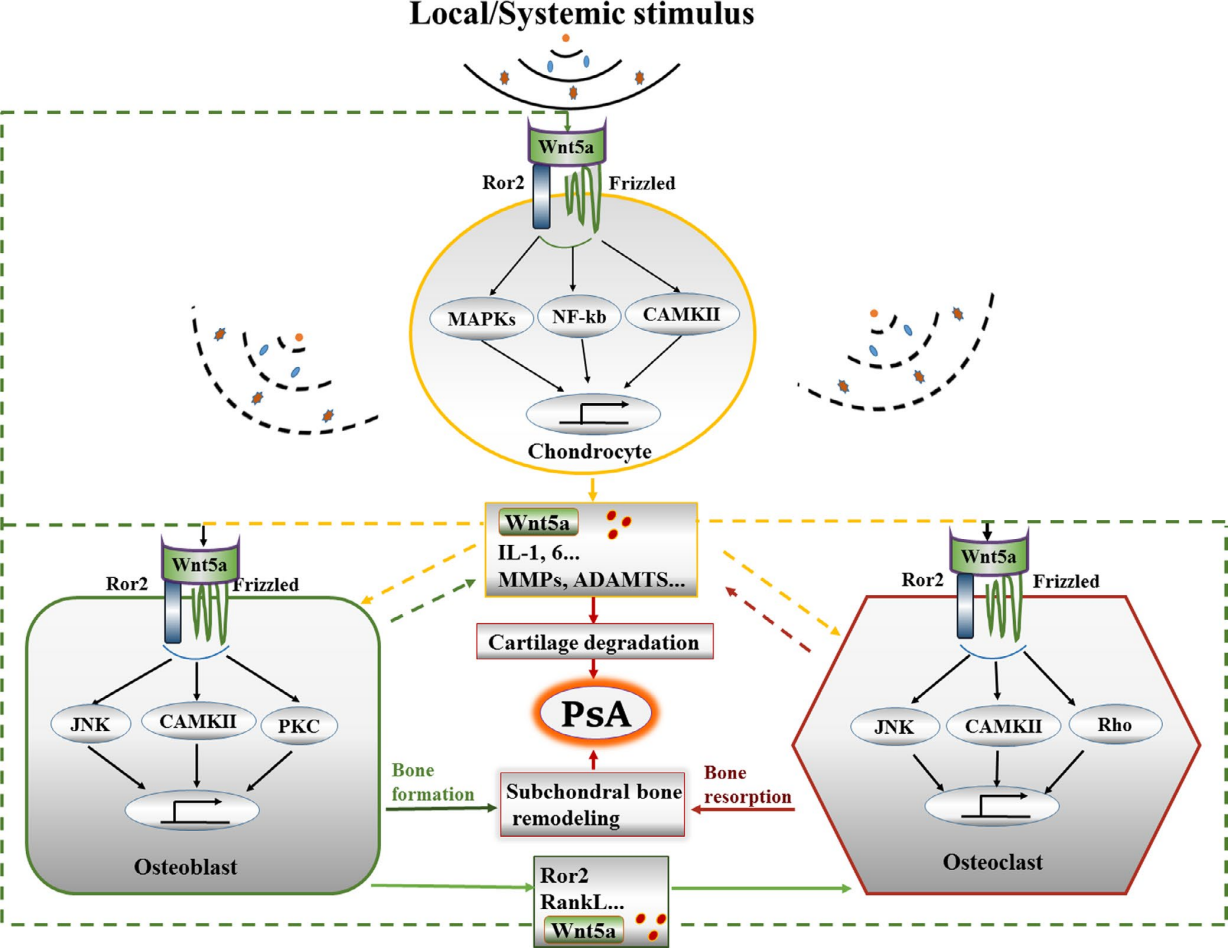
Model of the role and proposed mechanism of Wnt5a in psoriatic arthritis. Wnt5a produced by chondrocyte or osteoblast activated the non‐canonical signalling pathway and downstream cascades include CAMK Ⅱ, MAPKs, NF‐κB, JNK and/or PKC, Rho, thereby regulates the activity of chondrocytes, osteoblasts and osteoclasts, triggers both subchondral bone remodelling and cartilage catabolic metabolism, and finally lead to the development of psoriatic arthritis

These data support the hypothesis that Wnt5a plays a dual role, modulating bone remodelling as well as interacting with the immune system involved in psoriasis, and may thereby participate in the development of PsA. The Wnt5a–Ror2 signalling pathway regulates the activity of chondrocytes, osteoblasts and osteoclasts and is overexpressed in arthritis tissues. We therefore hypothesize that Wnt5a‐Ror2‐mediated interaction between the above‐mentioned cells triggers both subchondral bone remodelling and cartilage catabolism (Figure 2).

## 5 CONCLUSIONS

Based on the findings presented in this review, we propose that Wnt5a‐activated signalling pathways and other potentially interlinked factors mediate interactions among keratinocytes, immune cells and inflammatory factors, and that Wnt5a plays an important role in the development of PsO and PsA. However, the degree to which these responses in keratinocytes and leucocytes require Wnt5a remains uncertain. More research, particularly in vivo studies using exogenous Wnt5a inhibitors or conditional Wnt5a knockout in keratinocytes or other interacting cells, is needed to clarify the precise role and mechanism of the Wnt5a‐mediated immune response and inflammation in PsO and PsA. This will reveal whether Wnt5a is a ‘common checkpoint’ for PsO and PsA and, if so, would confirm Wnt5a as a potential target for the treatment of both PsO and PsA.

## CONFLICT OF INTEREST

The authors confirm that there are no conflicts of interest.

## AUTHOR CONTRIBUTIONS

FMT wrote much of the first draft, MTM provided suggestions and assistance in the writing, and ZXL guided the project and manuscript to its final form. All authors have read and approved the final manuscript.

## Data Availability

The datasets in the current study are available from the corresponding author on reasonable request.
